# PNS protects brain against ischemic injury by acting as an antagonist for AGE/RAGE signaling

**DOI:** 10.1002/ctm2.532

**Published:** 2021-10-21

**Authors:** Chunguo Wang, Xinqi Deng, Zheyi Wang, Shaonan Wang, Jinzhou Tian, Yaoyu Liu, Yize Sun, Biyuan Liu, Yuqing Wang, Canyu Su, Luhan Li, Ting Wang, Tao Lu

**Affiliations:** ^1^ Beijing Research Institute of Chinese Medicine Beijing University of Chinese Medicine Beijing China; ^2^ School of Life Sciences Beijing University of Chinese Medicine Beijing China; ^3^ Dongzhimen Hospital Beijing University of Chinese Medicine Beijing China; ^4^ School of Pharmacy Hebei University of Chinese Medicine Hebei China; ^5^ School of Traditional Chinese Medicine Beijing University of Chinese Medicine Beijing China; ^6^ The Third Affiliate Hospital Beijing University of Chinese Medicine Beijing China; ^7^ School of Chinese Materia Medica Beijing University of Chinese Medicine Beijing China

Dear Editor,

With a high mortality and morbidity rate, and the potential long‐term disabilities, stroke has been brought into attention in the medical field. Ischemic stroke accounts for 60%–70% of the total strokes seen in patients. However, current therapeutic approach is far from meeting the social and personal satisfaction on the quality of life. New approaches are urgently needed in this field. Seeking effective solution for diseases from Traditional Chinese Medicine has become an intriguing avenue with new perspectives for the development of drugs. Panaxnotoginsengsaponin (PNS) is a family of glycosylation molecules, being widely produced from *Araliaceae* herbs. The treatment of stroke with PNS complex is characterized by its rich composition, comprehensive efficacy, and minimized side effects.

By using the optimized LC‐ESI‐MS^n^ method, active components of PNS were investigated. Forty‐three saponins were identified according to multistage mass spectrometry (MS^n^) with retention time (Rt), high mass resolution and accurate mass measurements, with combination of consulting literatures (Figure S1, Table S1). The identification process of these 43 structures was illustrated on the basis of ginsenoside R1 (FigureS2). Potential regulations of PNS were illustrated with network pharmacology analysis which performed based on active components identified from PNS (Figure S3).

PNS’ protection against ischemic injury in MCAO/R brains was showed with TTC and TUNEL staining (Figure 1A–C, Table S2), while non‐significant changes between control and control+PNS groups was observed (Figure 1C). The protective roles of PNS were further studied with iTRAQ‐labeled quantitative proteomics technology combined with LC‐ESI‐MS^n^. A total of 4384 proteins and 18,647 peptides were identified and quantified with Proteome Discoverer 1.4 based on MS/MS data of iTRAQ labeling experiment (FDR < 0.01). Principal component analysis showed that protein expression variations caused by MCAO/R injury were successfully rescued by PNS treatment. A total of 91 differentially expressed proteins (*p* < 0.05, |log2fold change|≥0.26) were picked out for further study (Figure S4). Hierarchical clustering analysis showed a sharp contrast in regulation between model group and model+PNS group, suggesting a substantial role of the 91 differential proteins in biological functions (Figure 1D). By performing Causal network analysis with IPA (Ingenuity Pathway Analysis) (Figure 1E,F) as well as gene ontology analysis with DAVID database v6.8 (Figure 1G–I), regulations of AGE/RAGE signaling, which is seldom mentioned in saponins researches^1^, ^2^ and not enriched by network pharmacology analysis (Figure S3), played a critical role (Figure 1E,G). This suggested that AGE/RAGE signaling could be a new mechanism for PNS to exert its function. AGE refers to a heterogeneous group of glycated products, which act as RAGE ligand and participate a spectrum of chronic diseases.^3^ RAGE, a multi‐ligand transmembrane receptor, has been widely discussed. The RAGE/sRAGE expression in the brain is closely associated with the one in the plasma, representing a potential peripheral marker for stroke.^4^, ^5^, ^6^, ^7^ In this study, PNS showed considerable restraint on Caspase3 expression in brains of MACO/R rat (Figure 2A). Investigations was further given in PC12 cells with knock‐downed (SH‐RAGE) (Figure 2B,D) or RAGE over‐expressed (OE‐RAGE) (Figure 2C,E). The result showed that cleaved Caspase3 level is upregulated in OE‐RAGE, but suppressed in SH‐RAGE. Cleaved Caspase3 were significantly downregulated upon PNS treatment (Figure 2F–H), suggesting that PNS suppresses cell apoptosis by inhibiting function of RAGE. However, neither the sRAGE in serum (Figure 2I), nor the RAGE in brain samples (Figure 2J) were found being significantly altered under PNS treatment. These results indicated that PNS is less likely to influence RAGE signaling via regulating the level of sRAGE or RAGE.

**FIGURE 1:**
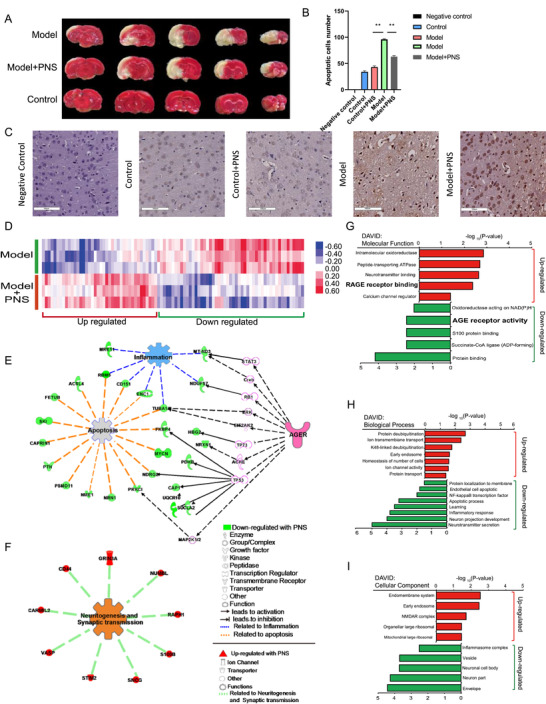
(A‐C) Protective effect of PNS against ischemic brain injury. (A) TTC staining of brains in Control, Model and Model+PNS groups (The brain was cut into five sections with average length, pale‐colored = ischemic area, red color = normal tissues). (B) The apoptotic cells number of different groups. Data are presented as mean ± SEM, *t‐*test, *n* = 6, **p < 0.05. (C) TUNEL staining of Negative control without TdT enzyme, as well as of brains in Control, Control+PNS, Model and Model+PNS groups, Scale bars = 100 μm. (D) Proteomics analysis of brain tissue. Heat map shows hierarchical cluster analysis and change trend of 91 significantly regulated proteins among control, model, and model+PNS groups. (E and F) IPA Causal network analysis based on protein‐sets of different regulations. (E) Down‐regulated proteins which involved in apoptosis and inflammation related functions; and AGE receptor is predicted as an upstream regulator. (F) Upregulated proteins involving in functions of neuritogenesis and synaptic transmission. (G‐I) GO analysis with DAVID database. Molecular functions (G), biological processes (H), and cellular components (I) were enriched based on 91 significantly regulated proteins among control, model, and model+PNS groups

**FIGURE 2:**
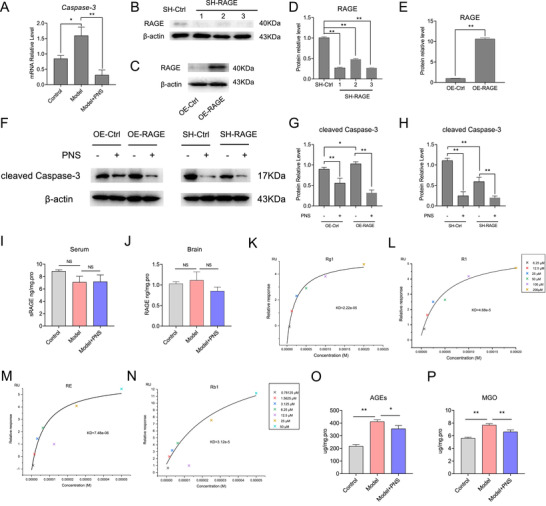
(A) *Caspase‐3* mRNA levels in vivo. (B‐E) Western blot verifications of three RAGE shRNAs knocking down (SH‐RAGE) (B and D) and RAGE overexpress (OE‐RAGE) (C and E) in PC12 cells. (F‐H) Expressions of cleaved Caspase‐3 in OE‐RAGE and SH‐RAGE PC12 cells responding to PNS administration. Data are presented as mean ± *SD*, *t*‐test, *n* = 3, *p < 0.1, **p < 0.05. (I) sRAGE level in serum. (J) RAGE level in brain. Data are presented as mean ± SEM, *t‐*test, *n* = 6, *p < 0.1, **p < 0.05. (K‐N) SPR analysis showing the interaction between notoginsenoside Rg1 (K), R1 (L), RE (M), Rb1 (N) and extracellular RAGE. (O) AGEs level in brain. (P) MGO level in brain. Data are presented as mean ± SEM, *t*‐test, *n* = 6, *p < 0.1, **p < 0.05

To address the interaction of PNS with RAGE, we employed notoginsenoside R1 and ginsenosides RE, Rg1 and Rb1, the dominant components of PNS, and extracellular RAGE protein for SPR assay. The ligands showed bindings in concentration‐dependent manner and reached steady state in short time. With steady‐state fitting methods, the binding constants (KD) of Rg1, R1, RE, Rb1 to RAGE were 22.2 μM (Figure 2K), 46.8 μM (Figure 2L), 7.48 μM (Figure 2M), and 31.2 μM (Figure 2N), respectively, which demonstrated effective and competitive bindings of PNS to RAGE. We further compared the binding ability of PNS molecules with other RAGE ligands. It was reported that S100A6's binding constant on RAGE V domain is 13.5 μM (97% species).^8^ Two overlapping medial peptides (23–45 and 40–50) of HMGB1 showed 8–40μM binding constant on RAGE.^9^ The binding of vRAGE to both BSA‐AGE and Lys‐AGE was observed with 6.2 μM and 19 μM.^10^ Therefore, PNS molecules’ binding to RAGE is compatible to other ligands and being competitive. Moreover, the increased expression of AGEs in brains of MCAO/R rats was moderately reversed upon PNS administration (Figure 2O). Similar regulation was found on methylglyoxal, the precursor of AGEs more significantly (Figure 2P). In addition, PNS did not regulate the levels of others RAGE ligands including S100B, HMGB1, as well as amyloid β‐protein precursor (Figure S6). Taken together, PNS acts as an antagonist of AGE/RAGE signaling.

Moreover, regulation of PNS on AGE/RAGE signaling was evaluated in PC12 cell. Downstream molecules of AGE/RAGE signaling were tested and quantified with characteristic peptides. A total of 10 downstream molecules of AGE/RAGE signaling, including Smad2, Diaph1, Prkca, Jun, Stat3, Stat5a, Cdc42, Caspase3, Rac1, and Mapk1, which respond significantly to PNS treatment, were screened with LC–PRM/MS (Figure 3A–J and S7) and confirmed by rt‐qPCR (Figure 3K–T). Downregulations on these molecules further illustrated the neural protective effect of PNS against ischemic brain damage.

**FIGURE 3:**
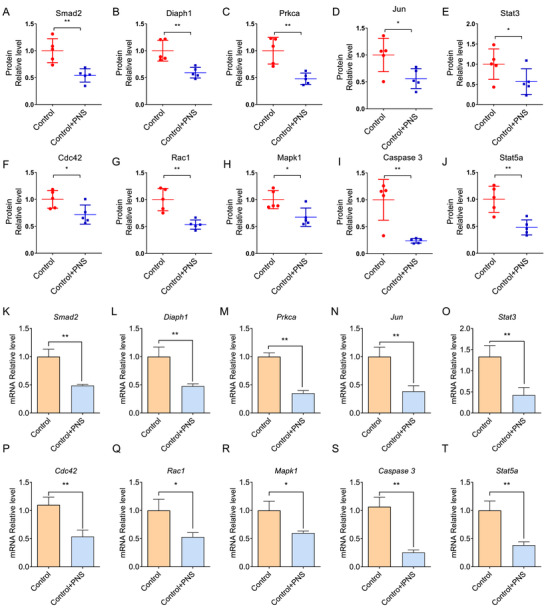
Protein expressions (A‐J) and mRNA levels (K‐T) of 10 target molecules in PC12 cell which are quantified with sub‐ions of characteristic peptide and screened by PRM. Data are presented as mean ± *SD*, *t‐*test, *n* = 5, *p < 0.1, **p < 0.05

**FIGURE 4:**
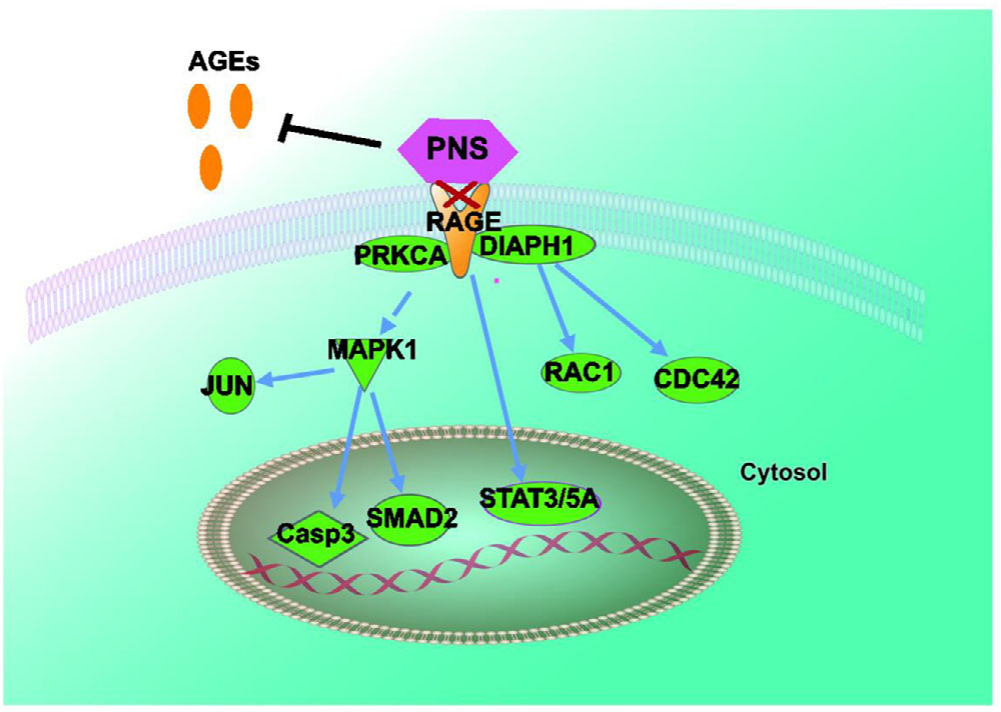
PNS negatively regulated AGE/RAGE signaling by competitive binding on RAGE as well as suppression on AGEs’ production

In conclusion, PNS protects brain against ischemic injury by acting as an antagonist for AGE/RAGE signaling (Figure 4. This study revealed the relationship of PNS and AGE/RAGE signaling pathway in stroke treatment. In stroke animal model, an unexpected role of PNS, a family of glycosylation molecules, was found to promote survival via negatively regulating AGE/RAGE signaling. This suggested a novel strategy for stroke intervention with specific glucoside molecules.

## CONFLICT OF INTEREST

The authors declare that there is no conflict of interest that could be perceived as prejudicing the impartiality of the research reported.

## Supporting information

Supporting Information

Supporting Information
