# Supplementary material for: PNS protects brain against ischemic injury by acting as an antagonist for AGE/RAGE signaling
Source: Clin Transl Med. 2021 Oct 21;11(10):e532. doi: 10.1002/ctm2.532 (PMC8530443; doi:10.1002/ctm2.532)
Supplement: Supplementary file 1 — Supporting Information [file CTM2-11-e532-s002.docx]

**Supplementary information**

**Reagents**

PNS complex was prepared in laboratory employing methods of refluxing extraction and macroporous resin-silica gel column chromatography purification[^1^](#_ENREF_1)^,^ [^2^](#_ENREF_2), and further processed to remove the impurity by referring to NO. WS-10460（ZD-0460）-2002 in Drug Standard of China. Standard substances of notoginsenoside R1, R2, ginsenoside Rg1, Rg2, Rg3, Rb1, Rb2, Rb3, Re, Rf, Rc, Ro, Rd, Rk1, Rh1, Rh2, ginsenoside F11 (content≥ 98%, Manst Biotechnology Co., Ltd, Chengdu, China). Methanol, formic acid and acetonitrile (Fishier Scientific). Trypsin (modified, sequencing grade, Promega). The other chemicals were purchased from Sigma–Aldrich (StLouis, MO) unless stated otherwise.

**Component identifications**

Identification process of the 43 structures in Table.S2 were illustrated on the basis of ginsenoside R1 as follows: Quasi-molecular ion peak was showed at m/z 931.52610[M-H]- with a retention time of 6.65 min. Characteristic fragment ions were shown at m/z 799，m/z 769，m/z 751，m/z 637，m/z 619，m/z 475，m/z 457, m/z 391 in LTQ-Orbitrap MS. The parent structure was speculated as a saponin, according to molecular formula of C47H79O18 (errors within 0.58 ppm), as well as typical ions and fragmentation patterns such as m/z 799, m/z 769, m/z 475 formed by the loss of a molecule of furanose, and two glucose. It was deduced that compound-4 was a triterpenoid saponins containing a molecule of furanose and ultimately identified as ginsenoside R1 with reference to the mass spectral fragmentation rule of ginsenoside R1 and consulting previous studies[^3-5^](#_ENREF_3).

**Animals**

Sprague-Dawley (SD) rats (Male, 40-50day old, 150 ± 10g) (Vital River Laboratory Animal Co. Ltd, Beijing, China). The experiments were performed in accordance with the Guide for the Care and Use of Laboratory Animals of Beijing University of Chinese Medicine with protocol approved by the Committee on Research Practice of Beijing University of Chinese Medicine (Approval ID: 2014BZHYLL0502, Approval date: 2014/05/02). After housed in polypropylene cages (5/cage) with unlimited access to food and water (12 h light–dark cycle, temperature of 24±2°C, relative humidity of 55%±5%) for 1 week, rats were randomly divided into control, control+PNS, model, and model+PNS groups (20/group).

*Control group:* At 0h, 3h, 6h after MCAO/R modeling, normal saline administration was performed via tail vein injection on rats without modeling.

*Control+PNS group:* At 0h, 3h, 6h after MCAO/R modeling, PNS administration (47.3 mg /kg body weight) was performed via tail vein injection on rats without modeling.

*Model group*: MCAO/R model was used in model group. For MCAO surgery, the rats were continuously anesthetized with isoflurane (1.5% of isoflurane/air (v/v)). Right MCA (Middle Cerebral Artery) was occluded with 4-0 silk suture. Reperfusion was performed at 1 h later, followed by normal saline administration via tail vein injection at 0h, 3h, 6h after MCAO/R modeling [^6^](#_ENREF_6).

*Model+PNS group:* At 0h, 3h, 6h after MCAO/R modeling, PNS administration (47.3 mg /kg body weight) was performed via tail vein injection on rats which were subjected to MCAO/R.

All specimens were obtained at 24h after reperfusion for further research.

**TTC and TUNEL staining**

After treatment of MCAO/R model mice with vehicle or PNS complex, the brain was sectioned coronally in 2-mm-thick, then stored in 2% TTC at 37℃ for 30min and 4% buffered formalin phosphate for 30min. TUNEL staining was performed following the protocol which is provided by manufacturer. The number of apoptotic cells were evaluated semi-quantitatively.

**Network Pharmacology analysis**

Chromatographic separation was performed on a Waters Acquity UPLC BEH-C_l8_ column (2.1×50 mm, 1.7 μm) at temperature of 30°C. High-resolution mass spectral analysis was performed on LTQ-Orbitrap mass spectrometer (Thermo Scientific, Bremen, Germany). Network pharmacology analysis was performed based on 17 active saponins identified in PNS. 91 target proteins were summarized by referring to literatures. The saponins-target proteins network diagram was presented using Cytoscape 3.6.1.

**Protein sample preparation**

Right hemisphere brain samples of three biological replications of each group were centrifuged at 16,000×g for 30 min at 4°C after homogenization and lysis. The supernatant was collected and stored in aliquots at 20°C. Following this, all the samples were digested with trypsin and processed in the FASP method which was described by Wisniewski et al[^7^](#_ENREF_7).

To label the tryptic peptides, centrifugal liquid was collected into a new tube and isopropanol was added. The labeling procedure was performed according to the manufacturer’s protocol, 113 tags for the control group, 117 tags for the model group, and 119 tags for the model+PNS group (Applied Biosystems, Foster City, CA).

**Tandem Mass Spectrometric (MS/MS) Analysis**

Freeze dried peptides were reconstituted in 200 μL of mobile phase A (pH 10.0, 2% (vol/vol) acetonitrile) and fractionated with a durashell-C_18_ column (4.6 × 200 mm, 5 μm, 300Å, PolyLC, Columbia, MD) on Prominence UFLC system (Shimadzu, Kyoto, Japan). Fractions were collected, vacuum-dried and reconstituted for LC-MS/MS analysis on an easy nano-flow HPLC system (Thermo Fisher Scientific, Odense, Denmark) coupled to a Q Exactive mass spectrometer equipped with Nano electrospray ion source (Thermo Fisher Scientific, Bremen, Germany). Purified peptides were separated on 50 cm C_18_ columns (an inner diameter of 75 µm, 1.8 µm beads, Dr. Maisch GmbH, Germany). Mass spectra were acquired in a data-dependent manner, with automatic switching between MS and MS/MS using a top-20 method.

**Protein Identification and Quantification**

The MS/MS spectra data were searched against the Uniprot KB/SwissProt Rat database of canonical sequences (December 2015, 22, 35719 entries) using automatic decoy database search engines via Proteome Discoverer 1.4 (Thermo Fisher Scientific). The false discovery rate (FDR) for both peptide and protein identification was set at 0.01[^8^](#_ENREF_8). After the identification of peptide and protein, iTRAQ datasets were analyzed and the abundance ratio of the proteins identified in model group vs. model+PNS group was used to assess the fold-changes. The quantitative value of each identified protein was determined by unique peptides only. Bias correction for unequal mixes in the different labeled samples was performed. All of the data was normalized by bias correction Normal distribution evaluation using the scatter plots by R.

**Bioinformatic data analysis**

PCA (Principal Component Analysis) was performed on global data with SIMCA-P 13.0 software (Umetrics AB, Umea, Sweden)[^9^](#_ENREF_9). Differential proteins (p<0.05, |log2fold change|≥0.26) were screened for bioinformatics analysis. Heatmaps was draw with the default clustering method of R (Euclidean distance)[^10^](#_ENREF_10). The open-source database DAVID was used for the analysis on cellular components, biological processes, and molecular functions[^11^](#_ENREF_11)^,^ [^12^](#_ENREF_12). IPA was applied for further investigation on responses to PNS treatment, including regulated pathways, networks, protein-protein interactions, functions and diseases[^13^](#_ENREF_13)^,^ [^14^](#_ENREF_14).

**Cell culture and CCK-8 assay**

PC12 cell line was obtained from SIBCB (Cell Bank of Shanghai Institute of Biochemistry and Cell Biology). By referring to previous reports[^15-17^](#_ENREF_15), RAGE-deficient PC12 cell line was produced with SHC201 vectors which were loaded with sequences of SH-RAGE1 (5' CTCTACGATCCCAATTCAA 3'; 5' TTGAATTGGGATCGTAGAG 3'), SH-RAGE2 (5' GGAAGCCGGAAATTGTGAATC 3'; 5' GATTCACAATTTCCGGCTTCC 3'), SH-RAGE3 (5' GGGCATTCAGCTGTTGGTTGA 3', 5' TCAACCAACAGCTGAATGCCC 3') and RAGE-overexpressed PC12 cell line was developed with PCDH vector which were loaded with sequences of OE-RAGE(5' GTTCCAGATTACGCTCTCGAG 3'; 5' ATTTAAATTCGAATTCTCGA 3'). Empty vector was applied as control.

Cells were cultured in Dulbecco’s modified Eagle’s medium (DMEM; Gibco) supplemented with 12.5% HS (Gibco), 2.5% FBS (Gibco), 100 μg/ml streptomycin (Gibco) and 100 U/ml penicillin (Gibco). 24 h after plantation, PC12 cells were treated with the presence or absence of 0.2μg/ml of PNS for 3h. Dosage of PNS on PC12 cells was decided according to CCK-8 assay. Cell Count Kit 8 (Beyotime, C0038) was used to evaluate cell proliferation ability. First, 2000 PC12 cells were seeded into 96 well plates and cultured with 100 μl complete culture medium. After 24 hr incubation, 100 μl 1640 medium containing PNS was added to each well. After 3 hr incubation, 100 μl DMEM containing CCK8 was added to each well. Multiskan™ FC (Thermo) was used to detect the value of OD450 at each well of the plates after incubation for 1 hr.

**PRM validation**

According to the quantitative proteomics result, 11 characteristic peptides of target proteins were selected for PRM validation. Orbitrap Fusion Lumos Tribrid mass spectrometer coupled with an EASY-nLC 1200 system (Thermo Scientific) was employed for PRM study. Raw data was processed in Skyline (version 19.1) to generate extracted-ion chromatograms and peak integration. The distribution of the relative intensities of multiple transitions, which is generated from the same precursor ion, should be in accordance with the theoretical distribution. The peak area of each characteristic peptide was obtained with Skyline analysis based on ＞4 sub-ions which are of high abundance and as consistent as possible on retention time in the secondary mass spectrometry. The results of PRM analyses were shown in supplementary files.

**Surface plasmon resonance (SPR) assay**

SPR was performed with Biacore 8K+ (GE Healthcare) instrument to determine the binding affinity of the major molecules of PNS, notoginsenoside R1 and ginsenosides Re, Rg1 and Rb1 with rat RAGE protein. By referring to previous reports[^18^](#_ENREF_18)^,^ [^19^](#_ENREF_19), recombinant rat RAGE protein (Gln24-Ala342, obtained from R＆D System), was immobilized to Series S Sensor Chip CM5 (GE Healthcare) with immobilization levels of ~12000 RU using standard coupling conditions in the active flow cells. HBS-N (10 mM HEPES, 150 mM NaCl, and 0.005% polyoxyethylenesorbitan (pH 7.4) with 2% DMSO was used as the running buffer. Binding response of ligand molecule to immobilized-protein surface was recorded. Data was collected under condition of contact time=60s, diss time=60s, flow rate=30μl/min, and solvent correction every 100 cycles within analysis. The maximum RU reflects comparative binding affinity and the dissociation constants (KD) is determined using the Steady-state affinity method.

**Western blotting and ELISA experiments**

Protein was obtained by applying RIPA Lysis Buffer (Beyotime, P0013B), protease and phosphatase inhibitors (Applygen, All-in-One, 100x) on cells or tissue samples. Membranes were incubated with cleaved Caspase3 (1:1000, Cell Signaling Technology, 9664), RAGE (1:2000, abcam, ab37647) and beta-actin (1:2000, Proteintech, 20536-1-AP) overnight. Images were developed by a chemiluminescence imager after horseradish peroxidase labeled secondary antibodies incubation. P-value was determined using Student's t-test, data represent mean ± SD. *P*-values of 0.1 or less were considered statistically significant.

ELISA experiment for detecting RAGE and sRAGE was performed with Rat SimpleStep ELISA Kit of RAGE (abcam, ab202409) according to product protocol. The levels of AGEs and MGO were detected with ELISA assay with Rat ELISA Kit of AGEs (Jiangsu Meibiao Biotechnology Co., Ltd, Jiangsu, China, MB-1811A) and MGO (Jiangsu Meibiao Biotechnology Co., Ltd, Jiangsu, China, MB-7228A). P-value was determined using Student's t-test, data represent mean ± SEM. *P*-values of 0.1 or less were considered statistically significant.

**rt-qPCR assay**

TRIzol (Invitrogen, 15596018) and TIANScript RT Kit (TIANGEN) were applied for RNA extraction and reverse transcription following the manufacturer’s instructions. 1 μg of total RNA was applied in cDNA synthesis with random primers and MMLV reverse transcriptase (Vazyme, R021). qPCR was performed in triplicate on CFX96 Real-Time PCR System (Bio-Rad). Data was calculated with the formula for relative FC=2^‐ΔΔCT^. P-value was determined using Student's t-test, data represent mean ± SD. *P*-values of 0.1 or less were considered statistically significant.

**Sequences**

| gene name | primer |
| --- | --- |
| stat3 | 5' AATACCATTGACCTGCCGAT 3' |
|  | 5' CATCGGCAGGTCAATGGTATT 3' |
| smad2 | 5' GGCTGAACTGTCTCCTACCAC 3' |
|  | 5' ATGTAATACAAGCGCACTCCC 3' |
| diaph1 | 5' AAGATTCCAAGGCAGAACCAC 3' |
|  | 5' TTGGCAACCTCTCCTGTCAGC 3' |
| prkca | 5' CCCTGCTCTACGGACTTATCCAC 3' |
|  | 5' ACTTCTGCCTGAGTTCCACGTT 3' |
| jun | 5' AGCGCCTGATCATCCAGTCCA 3' |
|  | 5' CTCCTGCTCGTCGGTCACGTT 3' |
| cdc42 | 5' ACGACCGTTAAGTTATCCACA 3' |
|  | 5' GATCAATTTGGGTCCCGACA 3' |
| caspase | 5' CACCCGGTTACTATTCCTG 3' |
|  | 5' GCATTGACACAATACACGG 3' |
| rac1 | 5' ATGCAGGCCATCAAGTGTGTG 3' |
|  | 5' CAGGTATTTGACAGCACCGAT 3' |
| mapk1 | 5' CCGCGCTACACTAATCTCTCG 3' |
|  | 5' TTCTCATGTCTGAAGCGCAGT 3' |
| stat5a | 5' CCAAGTCCCTGCTCAAGAACGA 3' |
|  | 5' GTTGTTCAGGATCTCGCCACT 3' |
| SH-RAGE1 | 5' CTCTACGATCCCAATTCAA 3' |
|  | 5' TTGAATTGGGATCGTAGAG 3' |
| SH-RAGE2 | 5' GGAAGCCGGAAATTGTGAATC 3' |
|  | 5' GATTCACAATTTCCGGCTTCC 3' |
| SH-RAGE3 | 5' GGGCATTCAGCTGTTGGTTGA 3' |
|  | 5' TCAACCAACAGCTGAATGCCC 3' |
| OE-RAGE | 5' GTTCCAGATTACGCTCTCGAG 3' |
|  | 5' ATTTAAATTCGAATTCTCGA 3' |

**Reference**

1. Suo JL, Shen F, Mi HL. Study on extraction of total panax notoginseng saponin. *Chinese Journal of Pharmaceutical Analysis*. 2011;volume 31:1197-1198(1192)

2. Chen H, Gan C, Gong Y. Advances in researches of extraction, separation, and purification technologies for total saponins of panax notoginseng. *Medicinal Plant*. 2019;10:1-4

3. Xiong Y, Chen L, Hu Y, Cui X. Uncovering active constituents responsible for different activities of raw and steamed panax notoginseng roots. *Frontiers in pharmacology*. 2017;8:745

4. Chen B, Wei Y, Wang D, Jia X. Metabolism of ginsenosides rk 3 and rh 4 from steamed notoginseng in zebrafish by ultraperformance liquid chromatography/quadrupole-time-of-flight mass spectrometry. *Archives of pharmacal research*. 2015;38:1468-1476

5. Xie Y-y, Luo D, Cheng Y-j, Ma J-f, Wang Y-m, Liang Q-l, et al. Steaming-induced chemical transformations and holistic quality assessment of red ginseng derived from panax ginseng by means of hplc-esi-ms/ms n-based multicomponent quantification fingerprint. *Journal of agricultural and food chemistry*. 2012;60:8213-8224

6. Tsai S-K, Lin S-M, Hung W-C, Mok MS, Chih C-L, Huang S-S. The effect of desflurane on ameliorating cerebral infarction in rats subjected to focal cerebral ischemia-reperfusion injury. *Life sciences*. 2004;74:2541-2549

7. Wiśniewski JR, Zougman A, Nagaraj N, Mann M. Universal sample preparation method for proteome analysis. *Nature methods*. 2009;6:359-362

8. Reiter L, Claassen M, Schrimpf SP, Jovanovic M, Schmidt A, Buhmann JM, et al. Protein identification false discovery rates for very large proteomics data sets generated by tandem mass spectrometry. *Molecular & Cellular Proteomics*. 2009;8:2405-2417

9. Wu Z, Li D, Meng J, Wang H. Introduction to simca-p and its application. *Handbook of partial least squares*. Springer; 2010:757-774.

10. Tiessen A, Cubedo-Ruiz EA, Winkler R. Improved representation of biological information by using correlation as distance function for heatmap cluster analysis. *American Journal of Plant Sciences*. 2017;8:502-516

11. Sherman BT, Lempicki RA. Systematic and integrative analysis of large gene lists using david bioinformatics resources. *Nature protocols*. 2009;4:44

12. Dennis G, Sherman BT, Hosack DA, Yang J, Gao W, Lane HC, et al. David: Database for annotation, visualization, and integrated discovery. *Genome biology*. 2003;4:1-11

13. Krämer A, Green J, Pollard Jr J, Tugendreich S. Causal analysis approaches in ingenuity pathway analysis. *Bioinformatics*. 2014;30:523-530

14. Jiménez-Marín Á, Collado-Romero M, Ramirez-Boo M, Arce C, Garrido JJ. Biological pathway analysis by arrayunlock and ingenuity pathway analysis. *BMC proceedings*. 2009;3:1-6

15. Taxman DJ, Moore CB, Guthrie EH, Huang MT-H. Short hairpin rna (shrna): Design, delivery, and assessment of gene knockdown. *Rna therapeutics*. Springer; 2010:139-156.

16. Morales Torres C, Laugesen A, Helin K. Utx is required for proper induction of ectoderm and mesoderm during differentiation of embryonic stem cells. *PloS one*. 2013;8:e60020

17. Chen L, Chen L, Qin Z, Lei J, Ye S, Zeng K, et al. Upregulation of mir-489-3p and mir-630 inhibits oxaliplatin uptake in renal cell carcinoma by targeting oct2. *Acta Pharmaceutica Sinica B*. 2019;9:1008-1020

18. Thumb I. Fragment library screening against the hepatitis c drug target, ns5b 1b, in the search for novel allosteric drug leads.

19. Adihou H, Gopalakrishnan R, Förster T, Guéret SM, Gasper R, Geschwindner S, et al. A protein tertiary structure mimetic modulator of the hippo signalling pathway. *Nature communications*. 2020;11:1-10
