# Supplementary material for: PNS protects brain against ischemic injury by acting as an antagonist for AGE/RAGE signaling
Source: Clin Transl Med. 2021 Oct 21;11(10):e532. doi: 10.1002/ctm2.532 (PMC8530443; doi:10.1002/ctm2.532)
Supplement: Supplementary file 2 — Supporting Information [file CTM2-11-e532-s001.docx]

**Supplementary figures/tables**

**Fig.S1.** Total ion chromatogram (TIC) of PNS complex in negative ionization mode.

**Fig.S2.** Basis and process for identification of ginsenoside R1. **A.** MS^2^ spectrum. **B.** Cleavage behavior deduction based on quasi-molecular ion peak, characteristic fragment ions and retention time.

**Fig.S3.** Components-proteins network of 17 active saponins based on Binding DB database, PubMed database, and KEGG database (diagram drawn by Cytoscape 3.6.1).

**Fig.S4. A.** PCA on proteins in brain tissue of control, model, and model+PNS groups. **B.** Scatter plot showing 91 significantly changed proteins between model and model+PNS groups (marked in red, *p*<0.05, |log2fold change|≥0.26).

**Fig.S5.** CCK-8 assay of PNS on PC12 cells. Cell viability is 0.924 under 2ug/ml PNS.


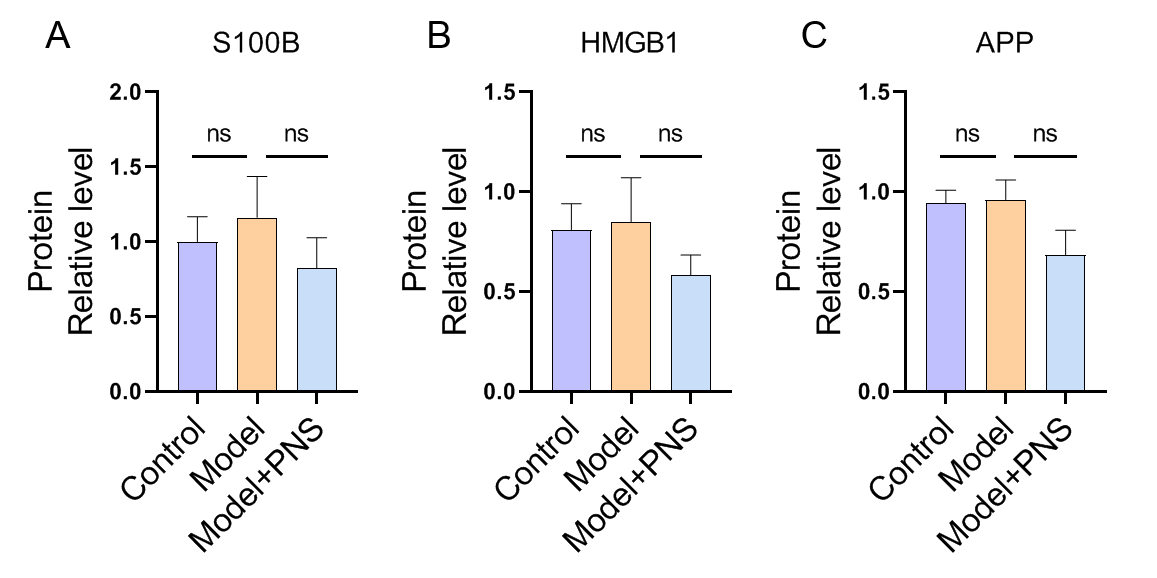


**Fig. S6.** Levels of RAGE ligands S100B (A), HMGB1 (B), and APP (C) changed slightly in brain. Data are presented as mean ± SEM, t-test, n=6.

**Fig. S7.A-J.** Intensity of sub-ions of each characteristic peptide which belongs to the 10 target proteins in Skyline.

**Tab.S1.** MS^n^ basis for identification of 43 saponins.

| NO. | t_R_/min | Molecular formula | Observed mass(*m/z*) | Calculated mass(*m/z*) | Mass accuracy(ppm) | (-)-ESI-MS/MS (*m/z*) | Identification |
| --- | --- | --- | --- | --- | --- | --- | --- |
| 1 | 10.35 | C48H82O18 | 946.55011 | 945.54229 | 0.58 | MS2:799；783；765；637；619；475 | Ginsenoside Re |
| 2 | 7.15 | C42H72O14 | 800.4922 | 799.48438 | 0.68 | MS2:637；475 | GinsenosideRf |
| 3 | 7.15 | C42H72O14 | 800.4922 | 799.48438 | 0.68 | MS2:799；637；619；475 | Ginsenoside Rg1 |
| 4 | 6.65 | C47H80O18 | 932.53446 | 931.52664 | 0.58 | MS2:799；637；475 | Notoginsenoside R1 |
| 5 | 9.53 | C42H72O13 | 784.49729 | 783.48947 | 0.7 | MS2:621；459 | 20(R)-Ginsenoside Rg3 |
| 6 | 9.69 | C42H72O13 | 784.49729 | 783.48947 | 0.7 | MS2:621；459 | Ginsenoside F2 |
| 7 | 9.6 | C54H92O23 | 1108.60293 | 1107.59512 | 0.48 | MS2: 945；783；621；459 | Ginsenoside Rb1 |
| 8 | 10.35 | C48H82O18 | 946.55011 | 945.54229 | 0.58 | MS2: 783；621；459 | Ginsenoside Rd |
| 9 | 9.53 | C42H72O13 | 784.49729 | 783.48947 | 0.7 | MS2: 621；459 | Ginsenoside Rg2 |
| 10 | 9.69 | C42H72O13 | 784.49729 | 783.48947 | 0.7 | MS2:603;585;471;423 | Ginsenoside Rg3 |
| 11 | 7.1 | C36H62O9 | 638.43938 | 637.43156 | 0.86 | MS2:637；475 | Ginsenoside Rh1 |
| 12 | 10.35 | C48H82O18 | 946.55011 | 945.54229 | 0.58 | MS2: 783；621；459 | Gypenoside XVII |
| 13 | 9.6 | C54H92O24 | 1124.59785 | 1123.59003 | 0.48 | MS2:1103；1077；961；799；637 | Notoginsenoside A |
| 14 | 7.25 | C48H82O20 | 978.53994 | 977.53212 | 0.56 | MS2:815 475 | Notoginsenoside E |
| 15 | 12.32 | C48H80O19 | 960.52938 | 959.52156 | 0.57 | MS2:797；635；473；389 | Notoginsenoside G |
| 16 | 6.21 | C47H80O19 | 948.52938 | 947.52156 | 0.58 | MS2:785；621；475 | Notoginsenoside H |
| 17 | 8.22 | C48H82O18 | 946.55011 | 945.54229 | 0.58 | MS2:783；621；459 | Notoginsenoside K |
| 18 | 22.48 | C48H82O19 | 962.54503 | 961.53721 | 0.57 | MS2:799；637；619；471 | Notoginsenoside M |
| 19 | 16.52 | C48H82O19 | 962.54503 | 961.53721 | 0.57 | MS2:799；637；619；471 | Notoginsenoside N |
| 20 | 15 | C41H70O13 | 770.48164 | 769.47382 | 0.71 | MS2:637； 475 | Ginsenoside R2 |
| 21 | 13.74 | C48H82O19 | 962.54503 | 961.53721 | 0.57 | MS2:799；637；475 | Notoginsenoside R6 |
| 22 | 21.45 | C42H72O16 | 832.48203 | 831.47421 | 0.66 | MS2: | Floralginsenoside A |
| 23 | 7.84 | C42H72O16 | 832.48203 | 831.47421 | 0.66 | MS2: | Floralginsenoside B |
| 24 | 5.14 | C42H72O15 | 816.48712 | 815.4793 | 0.67 | MS2:637；619；475 | Floralginsenoside E |
| 25 | 19.87 | C42H72O15 | 816.48712 | 815.4793 | 0.67 | MS2:637；619；475 | Floralginsenoside F |
| 26 | 23.09 | C41H70O13 | 770.48164 | 769.47382 | 0.71 | MS2:637；619；475 | Ginsenoside F3 |
| 27 | 9.69 | C53H90O23 | 1094.58728 | 1093.57947 | 0.49 | MS2:961；799；627；475 | Floralginsenoside P |
| 28 | 5.49 | C43H72O15 | 828.48712 | 827.4793 | 0.66 | MS2: | Vina-ginsenoside R2 |
| 29 | 6.37 | C47H80O19 | 948.52938 | 947.52156 | 0.58 | MS2:623 | Vina-Ginsenoside R6 |
| 30 | 16.23 | C48H82O19 | 962.54503 | 961.53721 | 0.57 | MS2:915；857；799；475 | majoroside F5 |
| 31 | 6.48 | C48H82O19 | 962.54503 | 961.53721 | 0.57 | MS2:915；857；799；475 | majoroside F6 |
| 32 | 19.87 | C42H72O15 | 816.48712 | 815.4793 | 0.67 | MS2: | Floralquinquenoside B |
| 33 | 3.88 | C42H72O15 | 816.48712 | 815.4793 | 0.67 | MS2: | Floralquinquenoside C |
| 34 | 19.87 | C42H72O15 | 816.48712 | 815.4793 | 0.67 | MS2: | Floralquinquenoside D |
| 35 | 3.88 | C48H82O20 | 978.53994 | 977.53212 | 0.56 | MS2:815；475 | Ginsenoside I |
| 36 | 6.37 | C42H72O14 | 800.4922 | 799.48438 | 0.69 | MS2: | GinsenosideIa |
| 37 | 23.09 | C36H62O9 | 638.43938 | 637.43156 | 0.86 | MS2:475;391 | Ginsenoside F1 |
| 38 | 6.21 | C41H70O13 | 770.48164 | 769.47382 | 0.71 | MS2:517;475 | Chikusetsusaponin L8 |
| 39 | 5.31 | C36H60O8 | 620.42881 | 619.421 | 0.87 | MS2: | Ginsenoside Rk3 |
| 40 | 5.49 | C42H70O12 | 766.48672 | 765.4789 | 0.72 | MS2:615;571 | (20E)-Ginsenoside F4 |
| 41 | 6.43 | C41H70O15 | 802.47147 | 801.46365 | 0.69 | MS2:639;553;477;391 | Floralginsenoside C |
| 42 | 23.48 | C42H74O16 | 834.49768 | 833.48986 | 0.66 | MS2:787;637;519 | Notoginsenoside J |
| 43 | 6.23 | C30H50O9 | 554.34548 | 553.33766 | 0.99 | MS2:783;621;459 | Notoginsenoside R10 |

**Tab.S2.** Infarct size of brains in control, model, and model+PNS groups.

| Group | N | infarct size (SI) |
| --- | --- | --- |
| Control | 6 | 0.0±0.0 |
| Model | 6 | 0.23±0.1^###^ |
| Model+PNS | 6 | 0.11±0.08^**^ |
